# Commentary: The Metamorphosis of the Hero: Principles, Processes, and Purpose

**DOI:** 10.3389/fpsyg.2019.01836

**Published:** 2019-08-07

**Authors:** Richard Wundrack

**Affiliations:** Department of Psychology, Humboldt-Universität zu Berlin, Berlin, Germany

**Keywords:** hero, heroic transformation, personality development, voluntary personality change, major life events, self-determination theory, basic psychological needs, whole trait theory

Concerned that the important issues discussed by Allison et al. may be lost to researchers who are used to a more moderate language, I would like to highlight the relevance of the article to social, developmental, and personality psychology. Notably, the heroic transformation, a blueprint for dramatic positive change, relates to voluntary personality change (Baranski et al., 2017) and personality changes due to major life events (Bleidorn et al., 2018). The three arcs of the heroic transformation, i.e., becoming sociocentric, autonomous, and growth-oriented, closely match the three basic psychological needs facilitating motivated behavior according to self-determination theory: relatedness, autonomy, and competence (Ryan and Deci, 2000, 2017).

What distinguishes the three arcs from the three needs are the presumed extent of their societal impact and that this impact is exclusively positive. The term “heroic” only applies if both conditions are met. However, societal impact is vague and a matter of degrees. Although circumstances may sometimes call for a true hero, more often, the cumulative acts of many individuals, whereby neither the acts qualify as heroic nor the people as heroes, change the world. For example, research on major life events suggests that people become more conscientious after their first job (Specht et al., 2011). Arguably, regular employment mirrors a minor heroic transformation due to training regimens. Although an increase in conscientiousness does not qualify as heroic, collectively, even such small changes have the potential for serious societal impact (Funder and Ozer, 2019). Consider the dramatic economic loss that would transpire if the generations of young adults entering the workforce would not become more conscientious. Such implications make the principles and processes of the heroic transformation relevant to all of psychology, not only those interested in heroism.

Cognitive evaluation theory and organismic integration theory, sub-theories of self-determination theory, suggest that needs are both (i) preconditions and (ii) outcomes of self-actualization (Ryan and Deci, 2000; Deci et al., 2013). As preconditions, needs are satisfied by others or experienced as such: we feel related, autonomous, and competent. That is, we feel connected to and loved by others; we experience our actions as voluntary and the locus of control lies within ourselves; and we feel like our actions (can) make a difference. As an outcome, these needs are satisfied in that we do relate more to others, can act more autonomously, and are more competent in our actions.

Need satisfaction can also be seen as a precondition and an outcome of the heroic transformation. As a precondition, it is the first step of a self-actualization toward heroism, as an outcome, it is the first step toward “other-actualization”: the ability to satisfy the basic psychological needs of other people by creating social contexts that allow them to self-actualize. Consider the rebel leader who unites the political opposition (satisfies others' relatedness) and organizes their actions (satisfies others' competence) to fight for the political change they desire (satisfies others' autonomy). Becoming the rebel leader may be a heroic transformation in its own right, but it is the community of rebels acting as one that brings about political change. Thus, the leader enables the masses to self-actualize in the political domain by satisfying their needs, i.e., the leader other-actualizes. Although not a single rebel may qualify as a hero, the transformation and the actions of the group may be heroic regarding their positive, societal impact.

Integrating personality structures, processes, and development is the declared joint goal of a large body of personality psychologists (Baumert et al., 2017). Among others, effort in this direction has been made by integrating whole trait theory and self-determination theory (Prentice et al., 2018). Whole trait theory conceptualizes personality traits in a bottom-up manner as the stable patterns found in the aggregation of momentary personality states. Moreover, traits can be explained in terms of the psychological mechanisms that bring about the different, individual states. Prentice et al. argue that we strategically change our state levels to satisfy our needs as outcomes. In line with the aforementioned brief review, state-level changes may further occur due to changes in need satisfaction as preconditions and because the “heroes” among us may change their state levels to satisfy the needs of others (other-actualization). For example, one may act agreeably to connect to others (need satisfaction as outcome), one may be able to act agreeably because one feels connected to others (need satisfaction as precondition), or one may act agreeably so others feel related and can act upon their satisfied need (need satisfaction for other-actualization). Ultimately, one's other-actualization is another one's self-actualization. Thus, having completed a full circle from self- to other-actualization and back, we can appreciate that our lives are interdependent and should be understood as such.

In conclusion, the principles and processes of the heroic transformation suggested by Allison et al. are closer to many psychologists' interests than is immediately apparent. Thus, any psychologist interested in motivation, personality development, and interventional research can find inspiration from their article. Moreover, in emphasizing the highly social nature of these issues, I wish to underscore that these issues can only be fully understood if we conceptualize the individual from the very beginning as psychologically embedded, engaged, entangled and extending in their (social) environment (Menary, 2010). Indeed, the bodily boundaries of an individual may not be their psychological boundaries. That this approach is a fruitful endeavor is reflected in the multi-disciplinary research surrounding social baseline theory (Coan and Sbarra, 2015), the convoy model (Antonucci et al., 2013) and dynamic systems approaches (Kuppens et al., 2010; Revelle and Condon, 2015; Sosnowska et al., 2019). These approaches concede to John Donne's verse that “no man is an island” (1624) and acknowledge the loss of information if individuals are conceptualized separately from their social environments. Then, the smallest unit of psychological research is not the person but the person *in situ*.

## Author Contributions

The author confirms being the sole contributor of this work and has approved it for publication.

### Conflict of Interest Statement

The author declares that the research was conducted in the absence of any commercial or financial relationships that could be construed as a potential conflict of interest.
